# Effects of Ionic Liquid, 1-Ethyl-3-methylimidazolium Chloride ([EMIM]Cl), on the Material and Electrical Characteristics of Asphaltene Thin Films

**DOI:** 10.3390/ma15082818

**Published:** 2022-04-12

**Authors:** Sundarajoo Thulasiraman, Noor Mona Md Yunus, Pradeep Kumar, Zayyan Rafi Kesuma, Nadia Norhakim, Cecilia Devi Wilfred, Teuku Muhammad Roffi, Mohamad Faizal Hamdan, Zainal Arif Burhanudin

**Affiliations:** 1 Department of Electrical & Electronic Engineering, Universiti Teknologi PETRONAS, Seri Iskandar 32610, Malaysia; sundarajoo_16000640@utp.edu.my (S.T.); zayyan_21001299@utp.edu.my (Z.R.K.); nadia_19000181@utp.edu.my (N.N.); 2 Centre of Innovative Nanostructures and Nanodevices, Universiti Teknologi PETRONAS, Seri Iskandar 32610, Malaysia; pradeep.hitesh@gmail.com; 3 Centre of Research in Ionic Liquids, Universiti Teknologi PETRONAS, Seri Iskandar 32610, Malaysia; mona.yunus@utp.edu.my (N.M.M.Y.); cecili@utp.edu.my (C.D.W.); 4 Department of Fundamental and Applied Sciences, Universiti Teknologi PETRONAS, Seri Iskandar 32610, Malaysia; 5 Department of Electrical Engineering, Universitas Pertamina, Jakarta 12220, Indonesia; teuku.roffi@universitaspertamina.ac.id; 6 Group Technical Solutions Department, PETROLIAM NASIONAL BERHAD, Kuala Lumpur 50088, Malaysia; faizalh@petronas.com.my

**Keywords:** sludge, asphaltene, ionic liquid, organic, transistor

## Abstract

Asphaltene is a component of crude oil that has remained relatively unexplored for organic electronic applications. In this study, we report on its extraction technique from crude oil tank bottom sludge (COTBS) and its thin-film characteristics when 1-ethyl-3-methylimidazolium chloride ([EMIM]Cl) ionic liquid (IL) was introduced as dopants. The extraction technique yielded asphaltene with more than 80% carbon content. The IL resulted in asphaltene thin films with a typical root-mean-square surface roughness of 4 nm, suitable for organic electronic applications. The thin films each showed an optical band gap of 3.8 eV and a sheet resistance as low as 10^5^ Ω/□. When the film was used as a conductive layer in organic field-effect transistors (OFET), it exhibited hole and electron conduction with hole (*µ_h_*) and electron (*µ_e_*) mobilities in the order of 10^−8^ and 10^−6^ cm^2^/Vs, respectively. These characteristics are just preliminary in nature. With the right IL, asphaltene thin films may become a good alternative for a transport layer in organic electronic applications.

## 1. Introduction

Asphaltene is one of the main components of crude oil. It is a complex compound with the ability to self-assemble into larger molecules. The self-aggregation of asphaltene molecules is caused by heteroatom association [1,2,3,4,5]. Self-aggregation increases the viscosity of the crude oil and hence reduces its flowrate in risers and pipelines. It can become sludge that clogs wells, flowlines, surface facilities and sub-surface formations. Moreover, the sludge also contains pollutants, such as phenols and heavy metals that, if disposed indiscriminately, may lead to severe environmental pollution. There are a variety of techniques available to dispose of the sludge, but at a significant cost to the industry as well as the environment [6]. Therefore, for an economical and sustainable future, it is imperative to explore new applications for the asphaltene-rich sludge.

Asphaltene’s molecular structure appears as hexagonal rings of carbon atoms surrounded by hydrogen, metals, impurities, and some functional groups. Several structures have been proposed and they differ depending on the geographic origin of the crude oil [7,8,9,10]. Adopting these molecular structures and by density functional theory (DFT) calculation, the bandgap of asphaltene can be calculated to be ~1.85 eV, with HOMO and LUMO levels of ~4.78 eV and ~2.94 eV, respectively. These characteristics may be suitable for use as a transport layer in applications such as organic electronics and photovoltaics. Such applications would be best realized if the asphaltene could be doped with dopants that could contribute to the electrical conductions.

Ionic liquid (IL) comprises cationic and anionic components. It can be designed to have a definite set of properties. As their chemical variety has grown, ILs have been further divided into many types, such as room-temperature ILs (RTILs), task-specific ILs (TSILs), and polyionic liquids (PILs) [11]. The IL has been used as novel solvents in organic synthesis, catalysis, electrochemistry, electrocatalysts, and chemical separation in the oil industry [12]. It has also been used as an asphaltene dispersant agent in the oil industry [13]. Due to its molecular structure, IL can also be used as a doping agent in organic electronic applications. Atabaki et al., doped PEDOT:PSS with an imidazolium type of ionic liquid. In his study, the resistance of the PEDOT:PSS was reduced by 1.7–1.8% after the IL doping had taken place [14]. While the literature on the use of IL to prevent self-aggregation is abundant, literature on its use as a dopant in organic electronics is still very much limited. In fact, the work reported here, i.e., IL-doped asphaltene thin film as the transport layer for organic thin-film transistor, is the first of its kind.

In this work, the extraction methodology of asphaltene from crude oil tank bottom sludge (COTBS) will be initially introduced. Then, the extracted asphaltene will be dissolved in a solvent, together with the IL, 1-ethyl-3-methylimidazolium chloride ([EMIM]Cl). The solution is then spin-coated onto glass or Si substrate to form IL-doped asphaltene thin films. It is also spin- coated onto a commercially available pre-patterned source and drain back-gated Si wafer to form a back-gated organic field-effect transistor (OFET). Crucial parameters from materials and electrical points of view were then measured and analyzed.

## 2. Materials and Methods

### 2.1. Asphaltene Extraction

COTBS was initially obtained from an oil refinery in Malaysia. Then, 5 g of the sludge was weighed and heated using a muffle furnace. The sludge was weighed every 10 min during the heating process until it reached a constant weight. Asphaltene in COTBS was precipitated using decane with a 1 gm: 30 mL ratio following the ASTM D6560 standard. A magnetic stirrer was used to stir the solution for 8 h. The asphaltene precipitated oil sludge was then obtained by vacuum filtration of the mixture. Using the Soxhlet apparatus, toluene was used to dissolve the asphaltene from the oil sludge. The cycle was repeated for an additional eight hours or until the solvent was colorless. Finally, the toluene was separated from the asphaltene solids using rotary evaporation. 

### 2.2. Preparation of Asphaltene and [EMIM]Cl Stock Solution

Two solutions were prepared separately as follows: dissolved asphaltene, which consists of 4 mg of asphaltene solids in 10 mL of toluene, and [EMIM]Cl, which consists of 13.86 mg in 10 mL of chloroform. The dissolved asphaltene solution is considered a pure asphaltene solution without dopant. For doping purposes, the two solutions were mixed and sonicated for 15 min. The mixtures were prepared by adding different concentrations: 1, 2, and 5 wt% of [EMIM]Cl for optical and surface morphology analysis. Furthermore, the doping ratio was increased in steps of 10 until 110 wt% of [EMIM]Cl for sheet resistance optimizations. 

### 2.3. Thin-Film Formation

Glasses used as substrates were initially washed in deionized water (DIW), acetone and isopropanol alcohol (IPA). The stock solution was then spin-coated onto the glass at 2500 rpm for 60 s to ensure a uniform coating by using centrifugal force. Then, the rotation speed was increased to 3000 rpm for 5 s to reduce the thickness of the film. Finally, the rotation was reduced to 500 rpm for 60 s before the process ended. The samples were then heated on a hot plate at 60 °C for 300 s to remove any remaining solvents. The prepared samples were used for optical, surface morphology analysis, and electrical characterization.

### 2.4. Elemental Analysis

Elemental analysis was carried out in the equipment modeled “Vario Micro Cube”. The asphaltene sample was weighed and loaded into the integrated carousel in a tin vessel. Before moving to the combustion tube, the sample was transferred to the ball valve and flushed with helium carrier gas to remove atmospheric nitrogen. At 1200 °C, catalytic combustion takes place by injecting oxygen directly into the sample through a lance. The second furnace was used for post-combustion. The mixture is then separated into its components using purge-and-trap chromatography and detected using a thermal conductivity detector after the combustion is reduced on hot copper. Based on stored calibration curves, a connected computer system calculates the element concentration from the detector signal and the sample weight.

### 2.5. Thin-Film Surface Analysis

The Nano Navi (E-Sweep) Atomic Force Microscopy (AFM) (Bruker, Billerica, MA, USA) was used to examine the surface morphology of the coated and uncoated glass samples. For all the samples, non-contact tapping mode was used with a 1.2 V constant operating force, 1 Hz scanning rate, and 0.1 µm Z-axis range to obtain the thin-film surface topographical profile. The surface area of 5.0 × 5.0 µm^2^ of the specimens were then evaluated for their root-mean-square (RMS) roughness and other parameters.

### 2.6. Functional Groups Analysis Using ATR-FTIR

Functional groups surrounding the asphaltene molecular structure were studied using Spectrum One modeled Pelkin Elmer equipped with diamond head Attenuated Total Reflection Fourier Transform Infrared Spectroscopy (ATR-FTIR) (PerkinElmer Ltd, Seer Green, Beaconsfield HP9 2FX, UK). Firstly, toluene was evaporated using a thermal bath to obtain the undoped and 1, 2 and 5 wt% of [EMIM]Cl-doped asphaltene solids. The sample holder was cleaned with acetone and the calibration was carried out prior to each sample measurement. Next, the solids were placed on the sample holder of the measurement equipment. Pristine [EMIM]Cl was also measured as a reference in this study. The 5 mg of each sample were placed on the sample holder. Then, the diamond head was lowered to be in contact with the surface of the samples. The samples were analyzed at ambient temperature from a collection of 16 scans per spectrum in the frequency range of 4000 cm^−1^ to 550 cm^−1^.

### 2.7. Sheet Resistance Measurement of the Asphaltene Thin Films

In this study, Lucas Labs S-302-4 Four Point Probe (4PP) (Signatone Corporation, Gilroy, CA, USA) is used for electrical characterization. The probes are spaced apart by a constant distance along a straight line. Two of the outer probes of the 4PP are used to supply current to the sample, while the other two inner probes are used to measure the resulting potential drops. The step size of the current is automated and derived based on the current that is pre-set into Keithley 2400 attached to the 4PP. 

Sheet resistance can be calculated using equation:

(1) $$R_{s}=4.53\frac{\Delta V}{I},$$

where *I* is the input current, Δ*V* is the voltage drop and 4.53 is the correction factor used in this measurement. The recorded sheet resistance of the film was averaged from five sheet resistance measurements obtained at different points on the film.

### 2.8. Optical Bandgap Determination from UV-Vis Spectroscopy

The UV-Vis spectra were obtained using CARY from Agilent Technologies. A hydrogen and a tungsten lamp were used as a UV and visible light source, respectively. The scan rate used was 600 nm/min and the wavelength was swept from 190 nm to 900 nm. The reference material was an uncoated glass slide, while the target specimen was a film-coated glass slide. The light source would be split and deflected to the target specimen and reference holders using beam splitter optics. The amount of light that penetrated the specimen and the reference material would be measured and compared. 

The bandgap in this study was calculated using the equation:

(2) $$E_{g}=\frac{1240}{\lambda_{onset}},$$

where *λ_onset_* is the onset absorption and *E_g_* is the calculated bandgap [15]. The onset absorption was identified from the intersection of the linear fitting with the x-axis on the UV-Vis plot [16].

### 2.9. Fabrication of Organic Field-Effect Transistor (OFET)

Bottom gate bottom contact (BGBC) OFETs were fabricated by spin coating asphaltene solution doped with 90 wt% of [EMIM]Cl on the prefabricated transistor structure. The prefabricated transistor test structure consists of a heavily doped silicon substrate (gate electrode, p-type 10^−4^ Ω cm), thermally grown silicon dioxide layer (gate dielectric, 300 nm), and thermally evaporated gold electrodes (source and drain contacts, 70 nm gold on 2 nm chromium). The width of the transistor was fixed at 1000 µm with 30, 40, 50, 60, and 80 µm as channel length. 

### 2.10. Characterization of the OFET

The OFETs were characterized using Agilent Technologies B1500A Semiconductor Device Analyzer (manufacturer, city, state abbreviation, country). To determine the output characteristics, drain current vs. drain voltage (*I_d_-V_d_*) was plotted. The drain voltage (*V_d_*) is swept from 0 to 50 V, while the gate voltage (*V_g_*) is swept from 0 V to 50 V. The *I_d_-V_d_* curve was plotted for *V_g_* = 0 V, 20 V, and 40 V. For the transfer characteristics, *I_d_-V_g_*, *V_g_* is swept from −50 V to 50 V with *V_d_* varying from 0 V to 50 V. The electron mobility (*µ_e_*), hole mobility (*µ_h_*), threshold voltage (V_th_), *I_on_*/*I_off_* current and subthreshold swing (SS) were extracted from the linear and saturation region of the *I_d_*-*V_g_* curves based on the published standard and handbooks [17,18,19].

## 3. Results and Discussion

### 3.1. Asphaltene Physical Appearance and Elemental Analysis

Asphaltene consists primarily of carbon, hydrogen, nitrogen, sulfur, and oxygen as well as trace amounts of metals, such as vanadium, nickel, and iron. Typically, in an asphaltene molecule, the carbon atoms are arranged in several polyaromatic clusters with side aliphatic chains and other functional groups attached. Table 1 shows the elemental analysis of the asphaltene extracted from this project, i.e., for both thermally treated and untreated COTBS.

**Figure 1:**
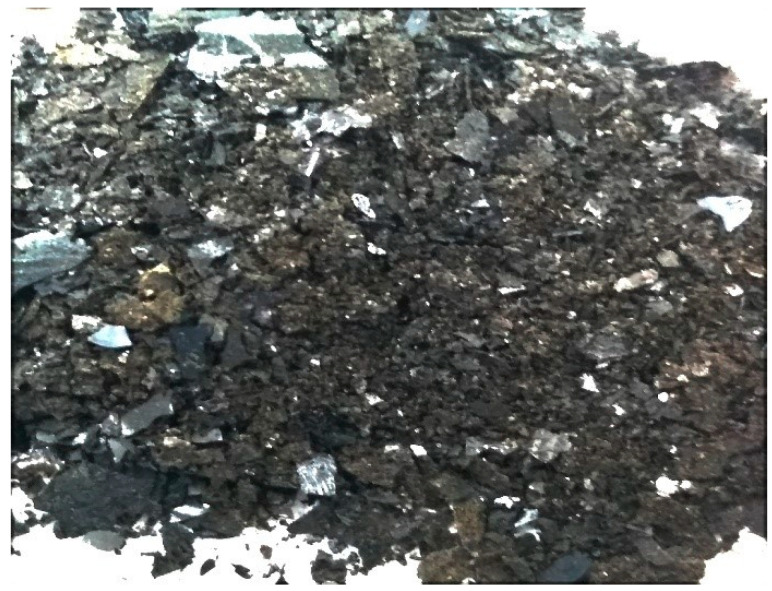
Black and shiny appearance of the asphaltene extracted from thermally treated COTBS.

Asphaltene extracted from the thermally treated COTBS shows a higher carbon content compared to the asphaltene extracted from the untreated COTBS. It is believed that heating the COTBS before asphaltene precipitation helps to remove moisture, thus reducing the number of hydrogens in the extracted asphaltene samples. Contents of nitrogen and sulfur also increased from the effect of thermal treatment of the COTBS. During thermal treatment, nitrogen was released from pyridine and indole composition, whereas sulfur was released from hydrogen sulfide bonding and sulfur oxide compounds originating from the COTBS. Nitrogen and sulfur in the asphaltene might present as surface functional groups at the edges of the asphaltene structure and this will influence the functionality of the asphaltene [20,21]. Asphaltene extracted in our study is black and shiny in appearance as shown in Figure 1.

### 3.2. Thin-Film Surface Analysis

**Figure 2:**
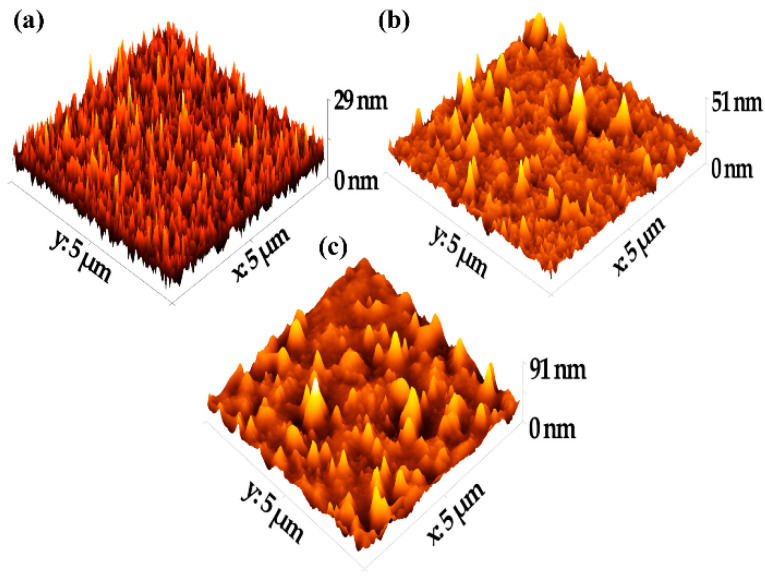
Typical 3D AFM images: (**a**) uncoated glass substrate; (**b**) 90 wt% of [EMIM]Cl-doped asphaltene thin film; (**c**) undoped asphaltene thin film.

The surface of a thin film significantly affects its mechanical and electrical transport properties. Hence, conducting surface analysis and assessing its roughness may have a tangible impact on the performance of electronic devices made from the thin film. Figure 2 shows typical 3D AFM images of asphaltene thin film on a glass slide for both doped and undoped asphaltene. A 3D AFM image of an uncoated glass slide is also included as a reference. 

The key parameters and the statistical analysis of the surface topography are summarized in Table 2. The surfaces were described using roughness parameters, such as the average surface roughness (S_a_), which is related to the average deviation of the surface irregularity from the mean line over one sampling length, and the root-mean-square surface roughness (RMS), which is calculated as the standard deviation of the surface height distribution and the peak-to-peak roughness. The RMS roughness of the three surfaces was 3.370 nm, 7.924 nm, and 3.758 nm for the blank glass slide, the undoped asphaltene film, and the 90 wt% of [EMIM]Cl-doped asphaltene film, respectively. It is apparent that the doped RMS roughness was much lower than the RMS roughness of the undoped asphaltene film. In fact, the RMS roughness of the 90 wt% of [EMIM]Cl-doped film was 3.758 nm, a value closer to the roughness of the uncoated glass slide, which was 3.370 nm.

Lower surface roughness for the doped film was caused by the lower viscosity of the asphaltene solution doped with 90 wt% of [EMIM]Cl. This modified the asphaltene structure in such a way that the intensity of the peak-to-valley decreased and smoothed the thin film surface, which resulted in a lower surface roughness compared to undoped asphaltene thin film [22,23].

Referring to the skewness and excess kurtosis definitions, these parameters describe the height symmetry of the surfaces. In our study, the skewness was 1.255 and 1.673 for undoped and doped samples, respectively. Thus, the peak distribution of the coating shows the right tail is longer than the left tail. In addition, the hills are dominant over the valleys, which indicates that the distributions are not perfectly symmetrical [24]. Values for excessive kurtosis are greater than 3 for both undoped and 90% [EMIM]Cl-doped asphaltene thin films, which indicate that both thin film surfaces are spiky and the distribution is leptokurtic [25].

In essence, the surface analysis shows that the asphaltene thin films have low surface roughness. The thin films have spiky surfaces, and the peak distributions are dominant over the valleys. More importantly, however, the ability of [EMIM]Cl to improve surface roughness of the thin films is established in this work. 

### 3.3. Functional Groups Analysis Using Attenuated Total Reflection Fourier Transform Infrared Spectroscopy (ATR-FTIR)

**Figure 3:**
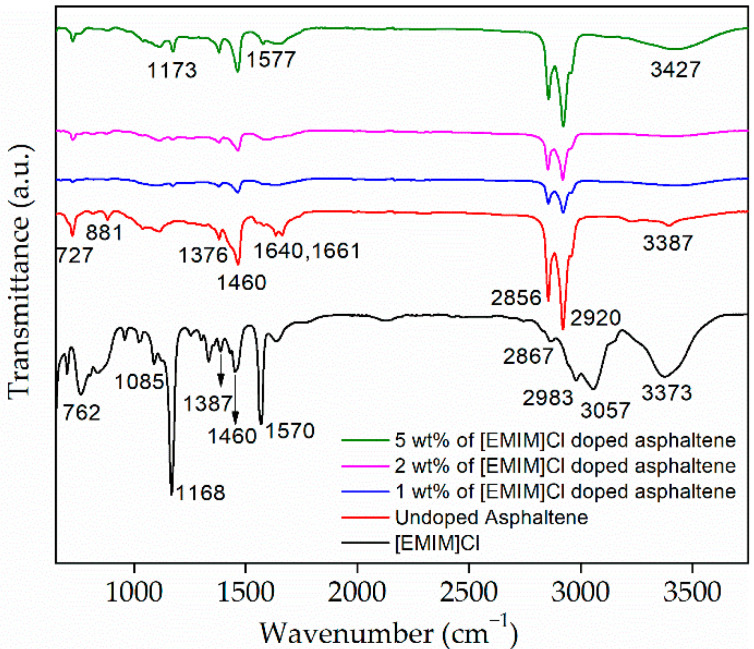
Fourier Transform Infrared spectroscopy of the undoped and [EMIM]Cl-doped asphaltene thin films.

Asphaltene molecular structure is commonly attached with functional groups. These functional groups were identified by using ATR-FTIR. Figure 3 shows FTIR spectra of asphaltene doped with 1, 2, and 5 wt% of [EMIM]Cl, respectively. As a comparison, spectra for undoped asphaltene and [EMIM]Cl are also included. There are a few peaks of interest that are visible in all asphaltene spectra. Particularly, we focus on the peaks at 2920 cm^−1^, 2856 cm^−1^, 1376 cm^−1^, 1460 cm^−1^, which were ascribed to C-H symmetric stretching, C-H asymmetric stretching, C-H symmetric bending, and C-H asymmetric bending, respectively.

In addition, for undoped asphaltene spectra, four adjacent aromatic C-H bonds and out-of-plane deformation vibration of one isolated aromatic C-H bond peaks were visible at 724 cm^−1^ and 881 cm^−1^, respectively. Furthermore, C=O or carbonyl stretching visible at the range of 1640 cm^−1^ to 1661 cm^−1^ and O-H or hydroxyl group stretching peak were observed at 3387 cm^−1^.

For [EMIM]Cl, the peaks at 762 cm^−1^ and 1085 cm^−1^ could be attributed to C-H out-of-plane bending and N-C bonding, respectively. Stretching of C-C vibration from the alkyl chain is observed at 1168 cm^−1^. The C-H symmetrical and asymmetrical bending and stretching for [EMIM]Cl is observed at 1376 cm^−1^ and 1460 cm^−1^, 2867 cm^−1^, and 2983 cm^−1^, respectively. The peak at 1570 cm^−1^ represents the symmetrical stretching of the N=C bond. In addition, the stretching of C-H of substituted polynuclear aromatics and hydroxyl group or O-H stretching appeared at 3057 cm^−1^ and 3373 cm^−1^, respectively [26]. The peaks and its associated descriptions are summarized in Table 3.

The plot shows the transmittance intensity for undoped asphaltene to be low at C-H symmetric and asymmetric, stretching and bending regions. However, as the first 1 wt% of [EMIM]Cl was introduced, the intensity increased significantly, implying the attachment of [EMIM]Cl to the functional group of the asphaltene [27]. More interestingly, as the [EMIM]Cl was increased to 5 wt%, the peak at 881 cm^−1^, ascribed to the out-of-plane deformation vibration of one isolated aromatic C-H bond, disappeared. At the same time, sharp and broadened peaks at 1173 cm^−1^ and 3427 cm^−1^ appeared. These peaks represent the non-hydrogen bonded stretching mode of C-OH groups [28] and O-H stretching, respectively. As the concentration of [EMIM]Cl increased, the peaks that represented the O-H group became more intense. These changes prove there were interactions between the hydrogen of the hydroxyl group and the chloride ions [29]. The FTIR analysis shows that IL doping can alter the asphaltene’s molecular structure.

### 3.4. Sheet Resistance (R_S_) Measurement of the Asphaltene Thin Films

**Figure 4:**
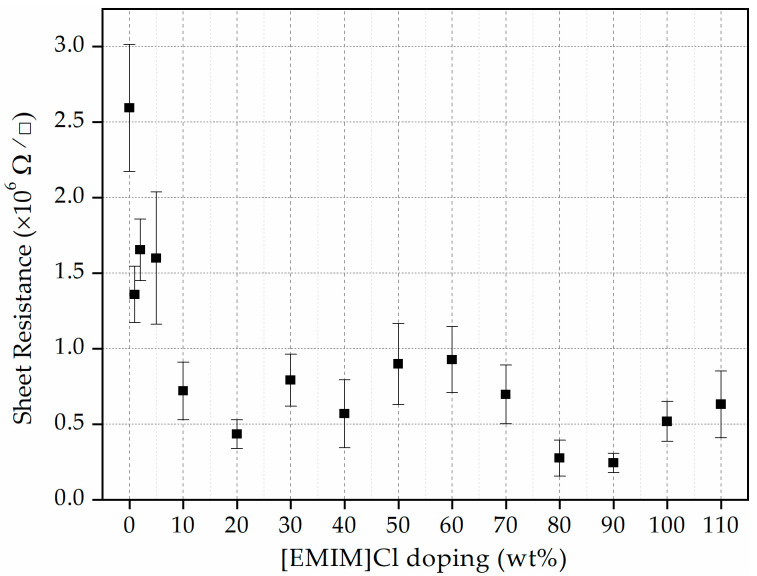
Sheet resistance of the asphaltene thin films as a function of [EMIM]Cl concentrations.

In our previous work, asphaltene extracted from thermally untreated COTBS showed R_S_ in the order of 10^10^ Ω/□ [30]. In this work, however, the R_S_ has been reduced significantly by the thermal treatment. Without ionic liquid doping, the R_S_ has already dropped by four orders of magnitude down to ~10^6^ Ω/□. More interestingly, with the introduction of [EMIM]Cl, the R_S_ drops further. Figure 4 shows the measured R_S_ versus the concentration of [EMIM]Cl used to dope the asphaltene film. For [EMIM]Cl less than 10 wt%, the R_S_ is still in the order of ~10^6^ Ω/□. A further increase in the concentration of the [EMIM]Cl, on the other hand, shows on average a further one order of magnitude drop to ~10^5^ Ω/□. 

Sheet resistance is inversely related to the mobile charge carrier density and carrier mobility. In this case, the number of mobile charge carriers increases when the doping concentration of [EMIM]Cl is increased. This is evidenced by the reduction of the sheet resistance of the thin film. This scenario is true when the doping concentration is varied from 1–20 wt%. From 20–60 wt%, however, the sheet resistance increases slightly, and this is probably due to the steric crowding of ions, which creates a barrier in carrier motion and effectively reduces its mobility. Then, for doping concentration between 60–110 wt%, again the sheet resistance appears to decrease. This is probably due to the dissociation of the formed polyionization, which increases the number of ions and hence reduces the sheet resistance once again [31]. Similar observations were made in the study conducted by Singh et al., where the conductivity of the IL-doped chitosan polymer thin film shows a non-monotonic relationship for doping concentration at 0–250 wt% of 1-ethyl-3-methylimidazolium thiocyanate [32]. 

In addition, in the study conducted by Dobbelin et al. [33], IL doping of the PEDOT:PSS thin films helps in increasing the conductivity of the thin films. It has been demonstrated that the conductivity of the [EMIM]Cl-doped PEDOT:PSS film improved from 1 S cm^−1^ [34] to 55 S cm^−1^ at an optimum doping level of 57 wt% of IL. Lower conductivity of PEDOT:PSS resulted from the insulating PSS content and lack of dense packing of PEDOT chains. Adding the IL helps to reorganize the molecular structure of PEDOT itself and improve the conductivity. Based on a similar argument, it is concluded that IL doping could also improve the conductivity (or lower the sheet resistance) of the asphaltene thin film to an extent, as observed in Figure 4.

### 3.5. Optical Bandgap Determination from UV-Vis Spectroscopy

**Figure 5:**
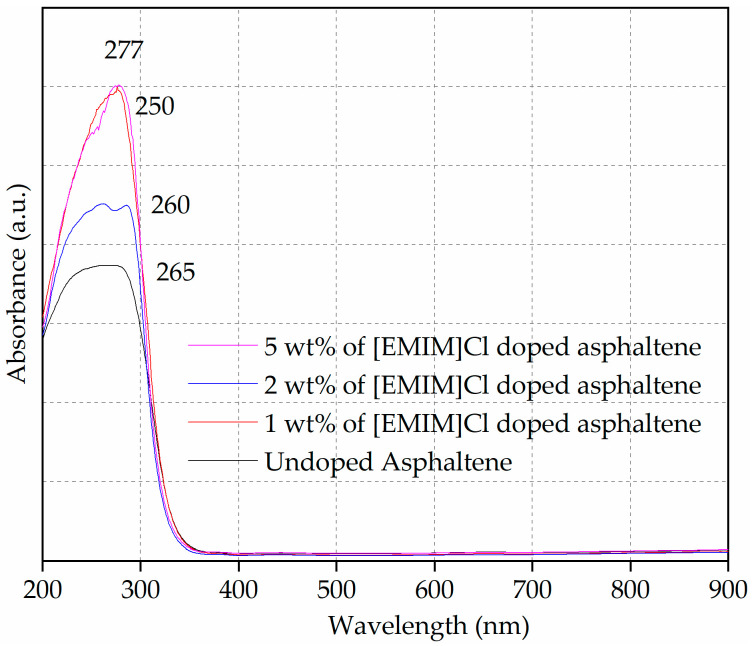
UV-Vis spectra of undoped and [EMIM]Cl-doped asphaltene thin film on glass substrate.

The optical properties of the undoped and [EMIM]Cl-doped asphaltene were investigated in solid-state form. The absorption spectra of the thin films are shown in Figure 5. The absorbance intensity in the UV range (200 nm–320 nm) indicates the presence of aromatic groups in the undoped and [EMIM]Cl-doped asphaltene film [35]. The profile of the undoped asphaltene film was rather broad compared to the [EMIM]Cl-doped thin films. The absorption spectra of undoped and 2 wt% of [EMIM]Cl-doped asphaltene film exhibited maximum wavelength (λ_max_) at 265 nm and 260 nm, respectively. Whereas, for the 1 and 5 wt% of [EMIM]Cl-doped asphaltene film, the λ_max_ was at 275 nm. For 5 wt% of [EMIM]Cl, an additional hump was observed at 250 nm.

The onset wavelength (λ_onset_) was recorded at 331 nm, 328 nm, 324 nm and 322 nm for undoped, 1, 2, and 5 wt% of [EMIM]Cl-doped asphaltene thin film, respectively. Furthermore, by increasing the doping to 5 wt% of [EMIM]Cl, there was an enhancement of the shoulder band at 250 nm spectrum and this absorption peak arose from π-π* transition [36]. These optical properties are tabulated in Table 4.

The increase in the absorption intensity (or hyperchromic shift) of the doped thin films resulted from the characteristics of the B-bands in the UV-Vis absorption spectra of heteroaromatic compounds, such as pyrimidine, pyridazine, and pyrazine [37,38], and the functional groups from ionic liquid [EMIM]Cl doping [39]. Furthermore, the occurrence of the red shift in the λ_max_ for the IL-doped film was potentially caused by the increment in the aromatics cluster sizes [40]. 

For organic materials, the bandgap is defined as the minimum energy required to excite an electron from the highest occupied molecular orbital (HOMO) to the lowest unoccupied molecular orbital (LUMO). The λ_onset_ values show a blue shift, which causes the increment in the calculated optical bandgap. The increment in bandgap shows the decrease in the particle size. The doping of IL could decrease the asphaltene particle size and, for a smaller particle, the energy required for an electron to get excited is higher. The lattice parameters increase as the diameter of the particle decreases, which results in wider spaces between the bands. Therefore, to cross a bandgap of greater energy, shorter wavelengths were observed, which resulted in the blue shift of the wavelength. 

Singh et al., conducted a study on the relationship between the semiconductor nanomaterials bandgap and sizes. Both the simulation and experimental study show the bandgap increase with the reduction in the size of the semiconductor nanomaterials [41]. Similarly, the simulation study conducted by Beriso et al., showed the bandgap of the germanium nanostructure increase with the reduction of its size [42]. The UV-Vis analysis in this study shows the asphaltene itself contains aromatics, and that [EMIM]Cl doping reduces the particle size and aids in altering the molecular structure of the thin films so that π-π* transition can take place. 

### 3.6. Characteristics of the Asphaltene-Based Organic Field-Effect Transistor (OFET)

**Figure 6:**
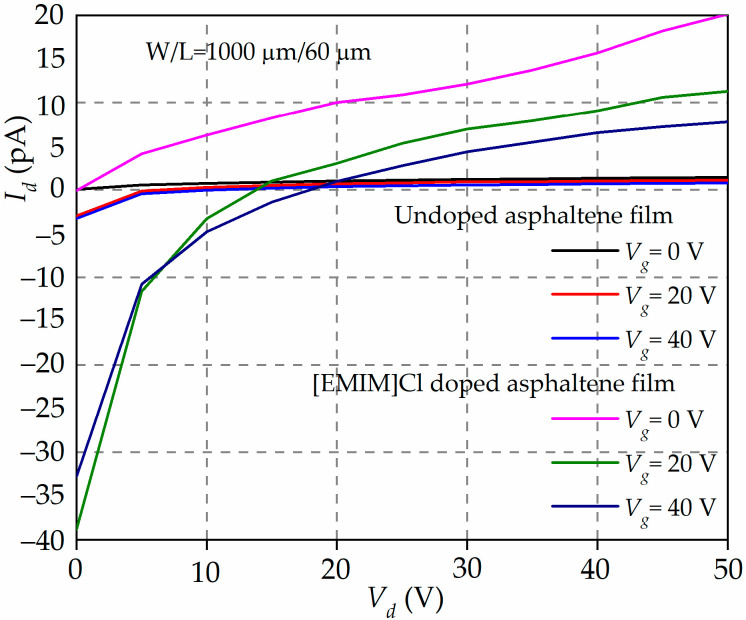
The output characteristics, *I_d_-V_d_*, of the undoped and [EMIM]Cl-doped asphaltene OFET bias at *V_g_* = 0, 20, and 40 V. The *I_d_* for [EMIM]Cl-doped asphaltene appears a few orders of magnitude higher than the *I_d_* for undoped OFET, except near the transition points where the *I_d_* change from negative to positive current.

Figure 6 shows the output characteristics, *I_d_-V_d_*, of OFETs with undoped and 90 wt% of [EMIM]Cl-doped asphaltene. The channel width and length of the OFETs are 1000 µm and 60 µm, respectively.

The most glaring feature of Figure 6 is that *I_d_* is small (in the range of a few tens pA). This is expected because the structure of the OFET has not been fully optimized, i.e., high-contact resistance, thick dielectric material, and high surface charge density. The next observation is that the *I_d_* flows even at *V_g_* = 0 V. This is typical for an OFET operated in depletion mode. It is also observed that for [EMIM]Cl-doped asphaltene OFET, the *I_d_* switches from negative to positive current at crossover voltage, *V_d_* ~15 V and *V_d_* ~20 V for *V_g_* = 20 and 40 V, respectively. The negative current can be ascribed to the current arising from the tunneling of carriers from the source to the gate contact. This is feasible considering that the electric field across the oxide is much greater than 10^6^ V/cm [43]. The *I_d_* becomes positive when the effective tunneling electric field has been reduced by the opposing electric field arising from the increase of the *V_d_*. More importantly, it is also observed that the *I_d_* for the [EMIM]Cl-doped OFET is two orders of magnitude higher than the *I_d_* from the undoped OFETs. The increase in *I_d_* is believed to be due to an increase in the number of charge carriers in the channel originating from the IL doping. 

**Figure 7:**
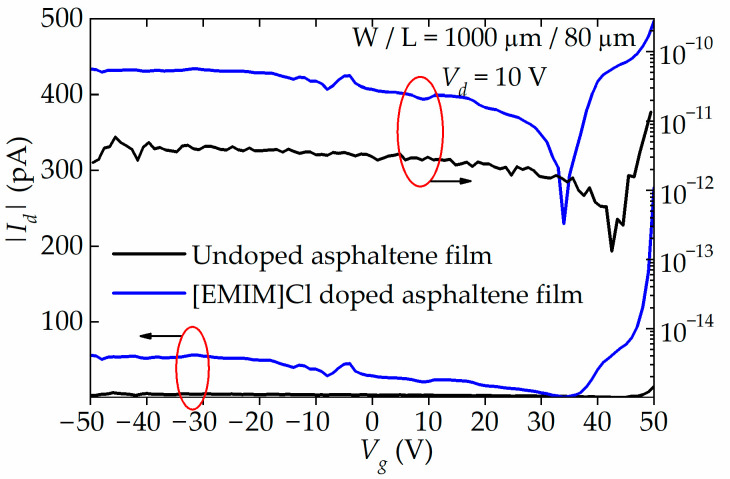
Transfer characteristics, |*I_d_|-V_g_*, of undoped and 90 wt% of [EMIM]Cl-doped OFET plotted in linear and logarithmic scale. Both hole and electron conduction are observed. The transition voltage from hole to electron conduction can be observed at 34.1 V to 42.8 V for doped and undoped OFET. The left-shift of the transition voltage suggests that the asphaltene thin film has been n-doped.

The transfer characteristics, *I_d_-V_g_*, of the undoped and doped OFETs plotted in linear and logarithmic scale are shown in Figure 7. From the plots, the *I_d_* for undoped OFET is small, within the range of 2–15 pA only. On the other hand, the *I_d_* for [EMIM]Cl-doped OFET is two orders of magnitude higher than the *I_d_* for the undoped OFET. The *I_d_* also exhibits hole and electron conduction, suggesting that the OFET has ambipolar transport characteristics. From the log plot, the transition voltage, where the change from hole to electron conduction occurs, is observed at 34.1 V and 42.8 V for doped and [EMIM]Cl-doped OFET, respectively. The left-shift of the transition voltage from 42.8 V to 34.1 V suggests that the asphaltene thin film has been n-doped by the [EMIM]Cl. Below these two transition voltages, the OFETs exhibit hole conduction, and it appears reaching saturation. Above the transition voltages, however, the OFETs exhibit electron conduction with *I_d_* limited to ~300 pA, simply because of the limitation of the instrument. With an optimized structure, the *I_d_* is expected to be much higher than the one observed in this work.

The *µ_h_* and *µ_e_* of the doped and [EMIM]Cl-doped OFETs were extracted from the *I_d_-V_g_* curve. The average value of the *µ_h_* and *µ_e_* for the undoped OFET are 4.89 × 10^−8^ cm^2^/Vs and 9.73 × 10^−7^ cm^2^/Vs, respectively. On the other hand, the average value of the *µ_h_* and *µ_e_* for the doped OFET are 9.45 × 10^−8^ cm^2^/Vs and 1.05 × 10^−6^ cm^2^/Vs, respectively. It is apparent that IL doping caused the *μ_e_* to increase by one order of magnitude while the *μ_h_* remained relatively unchanged. Furthermore, the threshold voltage (*V_th_*) and subthreshold swing (*SS*) also show good improvement for the IL-doped devices compared to the undoped devices, regardless of the channel length. As for the *I_on_*/*I_off_* ratio, in its current unoptimized OFET structure, it is only in the range of 10^0^–10^1^. The performance characteristics of the undoped and doped OFETs are summarized in Table 5.

In this study, asphaltene thin films extracted from thermally treated COTBS exhibited both hole and electron conduction. After IL doping, the *μ_e_* increases by one order of magnitude while the *μ_h_* remains relatively unchanged. The magnitude of the hole and electron mobilities are similar in 30 µm–80 µm channel length for BGBC architecture devices. On the other hand, the doping of the [EMIM]Cl shows some improvement in the surface roughness, on the extracted mobility and n-doped the asphaltene. 

## 4. Conclusions

In conclusion, asphaltene extracted from thermally treated COTBS contains a significant amount of carbon content compared to the untreated ones. As a thin film, it has a rather smooth surface. Doping the asphaltene with [EMIM]Cl led to an improved surface roughness with a typical ~4-nm RMS, making it suitable for organic electronic applications. The thin films showed an optical band gap of 3.8 eV and a sheet resistance as low as 10^5^ Ω/□. When the thin film was used as a conductive layer in OFETs, it exhibited ambipolar transport characteristics with *µ_h_* and *µ_e_* mobilities in the order of 10^−8^ and 10^−6^ cm^2^/Vs, respectively. These characteristics are just preliminary in nature. With the right IL and optimize device structures, asphaltene thin film may become a viable alternative as a transport layer in organic electronic applications.

## Figures and Tables

**Table 1:** Elemental Analysis of asphaltene extracted from thermally treated and untreated COTBS.

Elements	Thermally Treated COTBS (wt%)	Untreated COTBS (wt%)
C	81.76	64.22
H	8.91	9.83
N	1.31	0.59
S	1.43	0.68

**Table 2:** AFM surface analysis of the uncoated glass, undoped, and 90 wt% [EMIM]Cl-doped asphaltene thin film on glass.

Samples	RMS (nm)	Sa (nm)	Skew (S_sk_)	Excess Kurtosis
Glass	3.37	2.65	0.64	0.72
Asphaltene on glass	7.92	5.27	1.26	3.82
90 wt% [EMIM]Cl-doped asphaltene on glass	3.76	2.59	1.67	7.68

**Table 3:** Peak descriptions of the ATR-FTIR for the undoped and [EMIM]Cl-doped asphaltene.

Wavenumber (cm^−1^)	Description
724	four adjacent aromatic C-H
762	C-H out-of-plane bending
881	Out-of-plane deformation vibration of one isolated aromatic C-H
1085	C-N stretching
1168	C-C stretching
1173	C-OH non-hydrogen bonded stretching
1376, 1460	Methyl C-H symmetric/asymmetric bending
1661–1640	C=O (carbonyl) stretching
2856, 2920	Methylene C-H symmetric/asymmetric stretching
2867, 2983	Methyl C-H symmetric/asymmetric stretching
3057	N-H stretching of amide
3373, 3427	O-H stretching

**Table 4:** Maximum wavelength, onset wavelength and optical band gap for undoped and [EMIM]Cl-doped asphaltene thin films.

Asphaltene Film	λ_max_ (nm)	λ_onset_ (nm)	Optical Band Gap (eV)
Undoped	265	331	3.74
1 wt% of [EMIM]Cl	260	328	3.78
2 wt% of [EMIM]Cl	275	324	3.82
5 wt% of [EMIM]Cl	275	322	3.85

**Table 5:** Field-effect transistor characteristics of the undoped and [EMIM]Cl-doped asphaltene OFET.

Channel Length (µm)	Undoped Asphaltene OFET				[EMIM]Cl-Doped Asphaltene OFET			
	*µ_h_* (cm^2^/Vs)	*µ_e_* (cm^2^/Vs)	V_th_ (V)	SS (V/dec)	*µ_h_* (cm^2^/Vs)	*µ_e_* (cm^2^/Vs)	V_th_ (V)	SS (V/dec)
30	6.28 × 10^−8^	6.53 × 10^−7^	43.2	3.956	5.52 × 10^−8^	1.69 × 10^−6^	37.2	3.586
40	5.99 × 10^−8^	6.04 × 10^−7^	43.3	3.258	5.05 × 10^−8^	1.14 × 10^−6^	33.1	2.687
50	5.54 × 10^−8^	5.27 × 10^−7^	42.9	3.536	8.64 × 10^−8^	1.07 × 10^−6^	42.1	1.471
60	3.81 × 10^−8^	23.1 × 10^−7^	42.5	3.569	11.0 × 10^−8^	0.31 × 10^−6^	33.8	2.415
80	2.83 × 10^−8^	7.65 × 10^−7^	42.9	3.577	15.4 × 10^−8^	1.02 × 10^−6^	34.0	3.072

## Data Availability

Not applicable.
